# Microfluidic and Microscale Assays to Examine Regenerative Strategies in the Neuro Retina

**DOI:** 10.3390/mi11121089

**Published:** 2020-12-09

**Authors:** Maribel Vazquez

**Affiliations:** Department of Biomedical Engineering, Rutgers, The State University of New Jersey, 599 Taylor Road, BME-219, Piscataway, NJ 08854, USA; Maribel.Vazquez@rutgers.edu

**Keywords:** micropatterning, micro-cell culture, on-chip devices, extracellular gradients, transplantation, stem cells

## Abstract

Bioengineering systems have transformed scientific knowledge of cellular behaviors in the nervous system (NS) and pioneered innovative, regenerative therapies to treat adult neural disorders. Microscale systems with characteristic lengths of single to hundreds of microns have examined the development and specialized behaviors of numerous neuromuscular and neurosensory components of the NS. The visual system is comprised of the eye sensory organ and its connecting pathways to the visual cortex. Significant vision loss arises from dysfunction in the retina, the photosensitive tissue at the eye posterior that achieves phototransduction of light to form images in the brain. Retinal regenerative medicine has embraced microfluidic technologies to manipulate stem-like cells for transplantation therapies, where de/differentiated cells are introduced within adult tissue to replace dysfunctional or damaged neurons. Microfluidic systems coupled with stem cell biology and biomaterials have produced exciting advances to restore vision. The current article reviews contemporary microfluidic technologies and microfluidics-enhanced bioassays, developed to interrogate cellular responses to adult retinal cues. The focus is on applications of microfluidics and microscale assays within mammalian sensory retina, or neuro retina, comprised of five types of retinal neurons (photoreceptors, horizontal, bipolar, amacrine, retinal ganglion) and one neuroglia (Müller), but excludes the non-sensory, retinal pigmented epithelium.

## 1. Introduction

Biomedical research has embraced microscale and microfluidic technologies to examine cellular behaviors and their responses to endogenous and therapeutic stimuli [1,2]. These include the differentiation of specialized cells, migration towards concentration gradients, and biochemical signaling induced by extracellular factors, among many others [3,4,5]. The scale and precision of microfluidics offer exceptional advantages for quantitative study within miniaturized biological structures, including microvascular beds, the interstitium, and extracellular matrix [6,7,8]. As a result, microscale systems with characteristic lengths of single, tens, or hundreds of microns are increasingly adapted in the Nervous System (NS) to target the behaviors of neurons, glia, and the matrixes in which they reside [9,10,11,12]. Moreover, microfluidics have elucidated complex mechanisms of NS cell and tissue function by enabling the design of multicellular structures, two- and three-dimensional modeling, and high throughput integration of biochemical analyses [13,14,15,16]. Most recently, microfluidics have become central to regenerative medicine, where the intersection of technology, biology, and clinical science has advanced contemporary therapies for genetic and degenerative NS disorders [17,18,19].

### 1.1. Characteristics of Microfluidics

Microfluidics have been classically defined as systems that manipulate and interrogate sub-microliter volumes (μL) of fluid within engineered structures of critical lengths less than 1000 microns (μm), or 1 millimeter (mm) [20]. This multidisciplinary field has fused key principles of physics and surface chemistry [21,22,23] with engineering processes of microfabrication [24,25] to create precisely-controlled systems on the scale of biological cells and anatomical structures [23,25,26]. Physical phenomena that dominate fluid behaviors at the microscale are distinct, and induce fluid responses that are significantly different from those classically-observed at the macroscale, i.e., with millimeter dimensions and above [27,28]. For instance, the relative influence of gravity is greatly reduced at the microscale, while the effects of surface forces are more dominant. Notably, these phenomena include, but are not limited to: (i) Laminar flow, where movement of fluids transmits dissipative forces proportional to the flow rate [29,30,31,32] (ii) Interfacial forces, were the attractive forces between molecules at the interface of two or more fluids are equal to zero [33,34,35] (iii) Capillary action, which enables fluids to flow within narrow spaces of porous materials without the assistance of external forces [27,36,37,38] and (iv) Electrokinesis, where an imposed electric field induces fluidic transport [39,40]. In addition, devices fabricated at the microscale provide the remarkable advantage of precise experimental validation against analytical models of molecular and convective transport [41,42], viscoelasticity [43,44,45], electrochemical dynamics [46,47] and many others [48,49,50,51,52]. These advantages have pioneered the development of single and multiplexed microfluidic channels [53,54,55], micropatterned substrate surfaces [56,57,58,59], and three-dimensional (3D) microfabricated structures [60,61] to examine localized cell behavior. Moreover, microfluidic “upgrades” of conventional bioassays, such as culture flasks and chambers, have significantly enriched abilities to examine cell behavior on the in vivo microscale.

### 1.2. Applications in the Nervous System

Microfluidic and microscale technologies has been applied to examine NS development and function in animal models from invertebrates to humans. The central nervous system (CNS) consists of the brain and spinal cord, responsible for higher cognitive function, learning, and memory. While, the peripheral nervous system (PNS) encompasses the nerves outside of the brain and spinal cord that connect to muscles and organs for sensory-motor function. In addition, primary senses act through the CNS to stimulate different centers of the brain that facilitate detection and processing. For instance, sound waves are collected by the cochlea of the inner ear, where the cochlear nerve carries auditory sensory information directly to the brain [62]. Odorant information is transmitted to the CNS via olfactory sensory neurons of the nasal epithelium [63,64]. Microviolli of taste bud cells send information via sensory cranial nerves directly to the thalamus for taste [65,66], and tactile sensory input is transmitted via the somatosensory cortex of the brain [67,68]. By contrast, vision is unique because the eye is simultaneously considered independent tissue and accessible brain due to its direct attachment through the optic nerve [69]. As such, the visual system is often studied as a distinct part of the CNS, comprised of the eye and its connecting pathways to the visual cortex.

**Figure 1:**
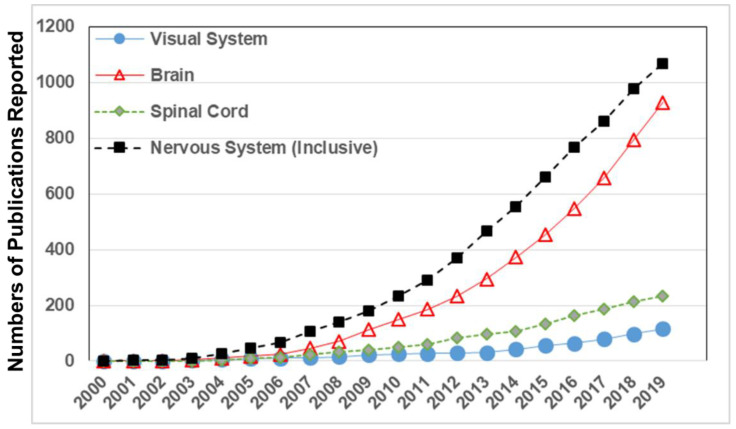
Numbers of published biomedical studies that applied microfluidic technologies to examine the nervous system (inclusive), brain (excluding visual system), spinal cord, and visual system. Data is gathered from publications reported in the Web of Science and National Library of Medicine (PubMed) each year from 2000 to date.

Recent entries in the databases of the Web of Science and National Library of Medicine (PubMed) demonstrate an exponential rise of projects applying microfluidic and microscale technologies to study the brain, spinal cord, and its neuromuscular and neurosensory components, as shown in Figure 1. These research efforts are commensurate with the increasing burden of neurological disorders reported worldwide, where the United States expended over $800B in the last year for treatments and care of NS disorders, such as Alzheimer’s disease [70], amyotrophic lateral sclerosis [71], traumatic brain/spinal cord injury [72,73] and vision loss [74,75]. Moreover, while the visual system has long been the domain of ophthalmology, visual health and its changes with age and chronic disease have become recent targets of many interdisciplinary studies in biomedical engineering and regenerative medicine [76,77,78,79].

### 1.3. Significance to the Visual System

The World Health Organization (WHO) estimates that an unprecedented 350 million adults will develop severe visual impairment by the year 2050 [80]. However, while much of the vision loss reported in middle income and under-developed nations is due to poor lens correction or injury [81,82], adults in higher income nations, such as in the United States and European Union, are more likely to experience progressive vision loss from chronic or degenerative cellular disorders related to metabolic syndromes and age [83,84]. These data beguile growing health disparities among the visually impaired, which depend upon non-medical factors, such as gender, age, and social economic status, to render vision loss a 21st century global health challenge [85,86].

**Figure 2:**
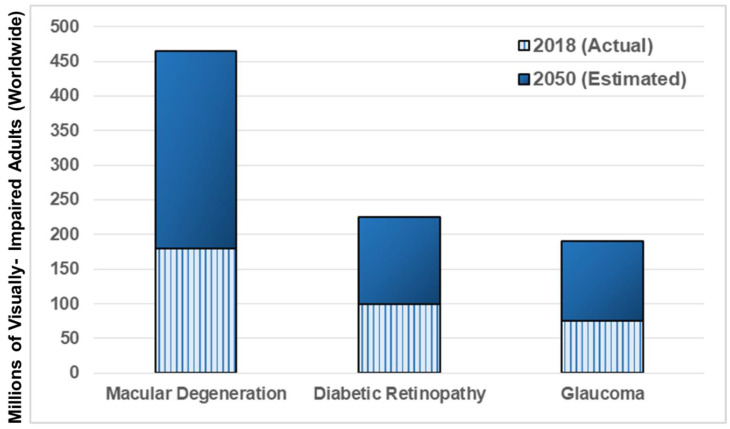
Numbers of adults diagnosed with the most prevalent retinal diseases, worldwide. Hashed bars reflect actual numbers of adults visually-impaired in 2018 with macular degeneration (wet and dry), diabetic retinopathy (proliferative and non-proliferative), and glaucoma (all subtypes). Solid bars denote estimated numbers of visually-impaired adults for each affected population by the year 2050, where the World Health Organization (WHO) expects to record dramatic increases (as per data from [80]).

Significant vision loss arises from dysfunction in the retina at the eye posterior, a photosensitive tissue connected directly to the brain through the optic nerve. Three retinal degenerative diseases constitute the majority of adult vision loss worldwide, as shown in Figure 2. These are macular degeneration, caused by the thinning of the retina [87,88], diabetic retinopathy, initiated by high glucose levels in the vasculature [89], and glaucoma, caused by elevated intraocular pressure [90]. Underlying biological causes of such degenerative vision loss include epigenetic and genetic factors [88,91], synaptic disruption of neural cells [92,93], cellular apoptosis [94], and detachment in different components of the eye [95] and its connecting pathways [96]. Natural processes of aging are also well-established to accelerate vision loss, highlighting the need for regenerative treatments that target the adult visual system [97,98,99].

### 1.4. Roles for Microfluidics in Neuro-Regenerative Medicine

Today’s regenerative projects combine stem-like cells and biomaterials with electro/physiochemical fields and biochemical factors to achieve tissue renewal. Microfluidic and microscale systems have modeled physiological scale and function across the NS [100], as well as helped elucidate biological processes of cell development, growth, collective response, synapse, and numerous others [101,102]. The manipulation of stem-like cells in microenvironments is central to regenerative medicine [103,104] as many projects seek to re-establish NS function by recapitulating key processes of development, e.g., regulated cell fate or specialized de/differentiation [105,106,107]. One exciting neuro-regenerative treatment is cell replacement therapy, where stem-like cells are transplanted into adult tissue to replace dysfunctional or damaged neurons [108,109,110]. Transplantation therapies have introduced cells with specialized bio-characteristics, and derived from various stem cell origins, into the adult retina of numerous animal models and humans [111,112,113,114]. Recent studies have additionally incorporated novel biomaterials and scaffolds to ease delivery of replacement cells and maintain cell viability in host tissue [115,116,117]. During treatment, surviving retinal stem-like cells must navigate tens to hundreds of microns to position within an existing cellular network, differentiate appropriately, and synaptically communicate with endogenous cells to re-establish the neuronal circuitry of vision [111,118,119].

Transplantation of retinal stem cells offers exceptional promise for restoring vision, as mature retinal neurons are unable to self-repair and hasten vision loss by propagating synaptic dysfunction via complex cellular interconnectivity. The retina is an excellent tissue target because its micrometer dimensions permit use of microliter volumes of reagents and lower numbers of replacement cells than other components of the NS [120,121]. Moreover, retinal transplantation sites are readily monitored via high-resolution, ophthalmic imaging to evaluate methods of cell delivery and cellular integration over time. While conventional bioassays have greatly elucidated fundamental cell behaviors of mammalian retina, microfluidic systems provide newfound opportunities to investigate localized effects of physiochemical factors, and apply external therapeutic fields to re-establish neuronal circuitry. Microfluidics and microscale assays offer three primary advantages for advancement of retinal replacement. First, is the analogous anatomical scale. Microsystems readily mimic both the retinal geometry and characteristic dimensions needed for quantitative and controlled studies at the scale of in vivo tissue. Second, is precision of the extracellular environment. Microfluidically-regulated stimuli can induce a variety of localized cell behaviors by manipulating ultra-minute concentrations of individual factors, including endogenous compounds and pharmacology. Experiments further correlate measured cell responses with the defined physiochemical properties of the microenvironment. Third, is real-time data acquisition. Microscale studies measure statistically-significant changes in cell responses over time to aid development and validation of emerging strategies using new cell groups, novel extracellular matrix molecules, and underexplored external stimulus fields. These advantages provide unique opportunities for microsystems to shepherd the success of retinal transplantation in adults, as microfluidic strategies facilitate controlled study of contemporary treatments in the visual system that are already applied in the brain, such as electro-modulation and pulsed electromagnetic fields. Moreover, microfluidics enable precise study of each cellular process required for stem cell transplantation, thereby, elucidating factors that aid the survival, differentiation, migration, and synapse of replacement cells, individually and in tandem.

Developmental biology has rapidly adapted the advantages of microfluidics to manipulate stem cell differentiation along pathways desired for regeneration. Recent projects have examined stem cell lineage progression via organoid models, i.e., simplified, microfluidic 3D organ culture systems that illustrate self-organized, cellular anatomy [122]. Contemporary retinal organoids have been derived from human pluripotent stem cells to resemble rudimentary optic vesicle-like structures with retinal layering similar to in vivo conditions [123,124]. These new models offer transformative opportunities to interrogate human development, disease progression, and gene therapy for treatment of genetic blinding diseases. However, developmental models provide significantly different physiochemical signals than adult tissue [125], making controlled study of stem cell responses to microfluidic cues from the adult retinal microenvironment critical for successful regeneration in mature and aging tissue.

### 1.5. Scope

The current article reviews microscale technology and microfluidics-enhanced bioassays developed to examine retinal cell behaviors in adult retina. This manuscript highlights technologies that leverage micrometer scales and (sub) microliter volumes to interrogate cellular responses critical to regeneration. MEMS (micro-electrical mechanical systems) technologies developed for implantable prostheses [126], independent of microfluidics, are excluded from this article. In addition, the selected literature is centered on the sensory retina of mammals, i.e., neuro retina, and does not include microfluidics or microscale systems developed for the retinal pigmented epithelium (RPE) [127] and retinal blood barriers [128,129]. Animal models of invertebrates and lower vertebrates (e.g., fish and avian) [130,131,132] were additionally excluded to highlight studies most translatable to humans and conserve manuscript length. References to contemporary reviews for each excluded area are denoted per section, as appropriate.

## 2. The Visual System

**Figure 3:**
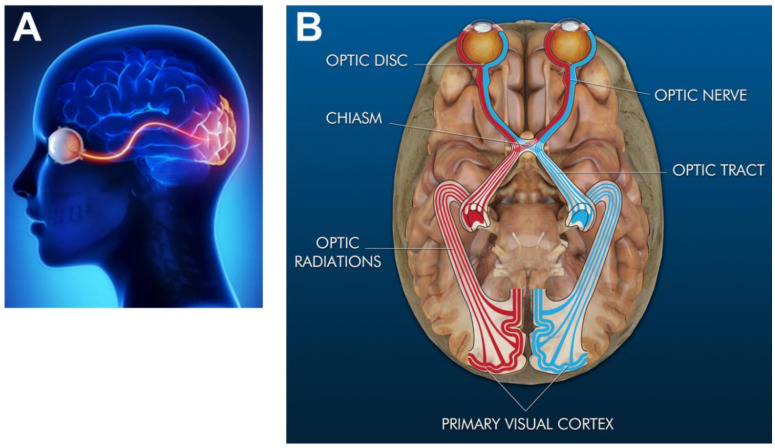
Simplified representation of the human visual system. (**A**) Sagittal plane (or side view) of the eye sensing organ and its connecting pathways to the visual cortex of the brain (highlighted in red) (Image reproduced with permission from The Discovery Eye Foundation). (**B**) Transverse rendering (or top view) of the primary components of the visual system overlaid onto the brain (Image reproduced with permission fromEyerudio Limited). The optic disc proceeds from the eye, where the axons of retinal ganglion exit to form the optic nerve. The optic nerve then connects to the optic chiasm, the X–shaped structure shown. The optic tract continues from the optic nerve and relays information to the optic radiations (or geniculocalcarine tract), which in turn transmit visual input to the primary visual cortex.

Vision is the detection and interpretation of light (defined as electromagnetic waves or photons) to produce images of objects in the brain. Light is absorbed and converted into electrochemical signals through the visual system, a part of the CNS comprised of the visual cortex, optic chasm, optic miasma, and the eye sensory organ, as per Figure 3 [133]. Mammalian eyes reside within orbital cavities of the skull that attach via extraocular muscles to assist eye movement for vision. The human eye has a 200-degree viewing angle and can detect over 10 million hues of color within the visible spectrum of 380 nm–740 nm wavelengths of light [134]. The eye is shaped as an imperfect sphere and is comprised of an anterior, or front segment, containing the cornea, iris, and lens, and a posterior, or back segment, composed of the vitreous, retina, choroid, and sclera [135]. The optic disc is the location where axons of retinal ganglion cells exit the eye to form the optic nerve. The disc is connected to the optic chiasm, the X-shaped structure formed by the crossing of optic nerves in the brain, as shown. The partial crossing of optic nerve fibers at the optic chiasm is highly significant to human vision because it permits the visual cortex to receive the same hemispheric visual field from both eyes. Superposition and parallel processing of these visual signals then allows the visual cortex to produce binocular and stereoscopic vision. The optic tract continues from the optic nerve to relay information from the optic chiasm to the geniculate nucleus. Lastly, optic radiations, also known as geniculocalcarine tracts, transmit visual input to the primary visual cortex.

### 2.1. Anatomy and Light Path

**Figure 4:**
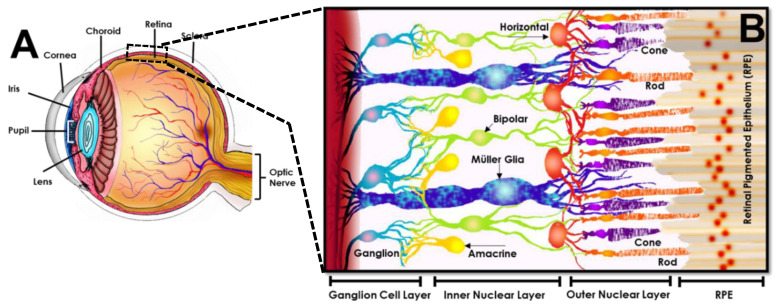
Representation of the human eye with retinal inset (image reproduced with permission from [143]). (**A**) Rendering of human eye illustrating (from anterior to posterior) the cornea that refracts light into the pupillary aperture controlled by the iris. Entering light is then focused through the lens and transmitted to a focal plane on the retina (dashed box), located at the eye posterior and connected to the optic nerve. The retina is adjacent to the choroid and the sclera, referred to as the “white” of the eye. (**B**) Cross-sectional, cellular schematic of the retina illustrating its three primary layers comprised of the ganglion cell layer (containing retinal ganglion cells), inner nuclear layer (hosting interneurons of amacrine, bipolar and horizontal cells), and outer nuclear layer (housing rod and cone photoreceptors). The sensory tissue, or neuro retina, is connected to the retinal pigmented epithelium (RPE), a non-sensory cell layer that interfaces with the outer blood retinal barrier at the eye posterior.

The path of incident light through the different components of the eye is shown in Figure 4. Light enters the eye anterior through the cornea [136], a transparent and avascular, dome-like structure that refracts incident light into the pupil, i.e., the black center of the eye. The pupil is surrounded by the iris [137], an annular and pigmented connective tissue that adjusts the pupil diameter to regulate the amount of light entering the eye. Entering light becomes incident upon the lens [138], a transparent and convex collagenous structure that changes its shape to transmit light as needed to create focused images of objects. The lens focuses transmitted light through the vitreous, a colorless, gel-like fluid that fills and supports the eye posterior [139]. The focal plane of transmitted light lies upon the retina, a photosensitive tissue hundreds of microns thick that lines the eye posterior [140]. The retina is directly connected to the brain via the optic nerve and contains networked cells that achieve phototransduction of light into the bio-electrochemical signals of vision. The retinal posterior contains the choroid [141], a highly fenestrated microvascular bed that supplies nutrients and oxygen, adjacent to the sclera, or “white” of the eye that provides its structural support and strength [142]. Of all components, the retina is singularly responsible for phototransduction and is considered the only neural tissue of the eye.

### 2.2. Retinal Tissue

Mammalian retina is a multi-laminated structure populated by an interconnected network of millions of neurons and glia, as per Figure 4. The retina includes a sensory portion, containing neural cells that process visual information, and a non-sensory portion comprised of a single layer of retinal pigmented epithelium (RPE). The RPE has become heavily-investigated for its critical neuroprotection of photoreceptors and regulation of transport across the blood retinal barrier. While, significant roles of the RPE in regenerative medicine lie outside of the current focus on sensory retina, bioengineering advances in RPE therapies are discussed in several excellent articles cited here [127,144,145].

The neuro retina is a laminated structure comprised of three layers of interconnected cells that communicate across two plexiform layers for signal transmission [120] (as per Figure 4). The outer nuclear layer (ONL) is bound by RPE cells on the retinal posterior and contains the cell bodies of rod and cone photoreceptor neurons. These photosensitive cells are known as primary neurons because they singularly achieve phototransduction of light. Rods and cones absorb light and change their membrane potential to synaptically transmit these signals across the inner plexiform layer to so-called secondary, or interneurons. Secondary neurons of the inner nuclear layer (INL) include horizontal, bipolar, and amacrine cells, each with multiple subtypes and with varying morphology for function. Horizontal cells are able to synapse directly with photoreceptors and transmit their signals to multiple surrounding bipolar neurons. Bipolar cells also synaptically connect directly with photoreceptors and are additionally able to receive signals from horizontal cells. By contrast, amacrine cells can only receive inputs from bipolar cells to synapse across the outer plexiform layer. Photonic signals from both amacrine and bipolar cells are transmitted across the outer plexiform layer to synapse with retinal ganglia of the ganglion cell layer (GCL). Axons from retinal ganglion cells form the optic nerve, which ultimately transmits photonic signals to the visual cortex to produce images of detected objects. In complement to the primary, secondary, and ganglion neurons of the retina, its tissue structure, function, and health are heavily regulated and supported by Müller glia. The cell bodies of these neuroglia reside within the retinal INL, while its cellular extensions span the full retinal thickness. The cells provide essential supporting and protective functions in the retina via uptake and regulation of neurotransmitters (e.g., glutamate, GABA), extracellular ions, growth factors, and pH [146]. Müller glia are also the principal neural cells that interact with the retinal vasculature, where they engage in processes to protect retinal neurons from injury or isolate functional circuitry from damaged or degenerated cells.

### 2.3. Visual Impairment and Loss of Vision

Millions of mature and aging adults experience progressive vision loss from retinal degeneration and injury, worldwide. The incidence of three primary retinal disorders alone is projected to increase four-fold by the year 2050 (as per Figure 2)**.** Age-related macular degeneration (AMD) is a visual impairment that causes loss of central but not peripheral vision over time. Fine details of image processing are progressively lost, irrespective of observation from close or afar. The blurred images caused by AMD worsen with time to severely impact vision and quality of life with age. AMD occurs at the interface between photoreceptors and the RPE to reduce oxygen supply, nutrient support, and maintenance of neurons and epithelium [147]. AMD pathology can be characterized as wet or dry, depending on the inclusion of effects from the vasculature. Dry AMD is the most common and less severe, where parts of the macula, or retinal center, thin with age concurrent with aggregation of drusen, i.e., deposits of extracellular waste comprised of proteins and lipids, such as cholesterol [148]. Wet AMD is less common, but more severe because it occurs via angiogenesis into retina. These abnormal vessels leak blood or other fluids to scar the macula and reduce vision. Subsequently, reduced perfusion and increased diffusion distances result in hypoxia, or low oxygen levels, to stimulate the release of pro-angiogenic cytokines and retinal angiogenesis. Pathological mechanisms of AMD remain incompletely understood, although related to processes of inflammation and oxidative stress [149], i.e., imbalance between cellular production and accumulation of oxygen reactive species that occur naturally with aging. Microfluidic technology has aided contemporary understanding of micro-aggregate formation and accumulation, models of pathological conditions, and advanced development of cell replacement therapies for AMD [129].

Diabetic retinopathy in adulthood affects three quarters of people diagnosed with diabetes, a chronic condition that alters the way the body processes glucose and/or produces insulin, the hormone that regulates blood sugar levels [150]. Diabetes is characterized by exceedingly high serum glucose levels and is often connected to dysregulation of insulin signaling. Elevated glucose levels cause damage to blood vessels that nourish the retina, and lead to retinal infiltration of new abnormal blood vessels that cause existing vasculature to swell, leak, or close to restrict retinal blood flow. Vascular changes in diabetic retinopathy are preceded by neurodegenerative processes that include hypertrophy and proliferation of Müller glia, partial loss of the retinal ganglion cell layer, and irregular thinning of the retina [151]. Over time fluids leak into the macula to deteriorate central vision in ways similar to wet AMD. The underlying reasons why retinal neurons become hyper-sensitive to elevated glucose remains incompletely understood, and are currently examined via microfluidic study of biological mechanisms.

Glaucoma is a group of neurodegenerative diseases where damage to the optic nerve leads to progressive and irreversible blindness via loss of peripheral vision and contrast sensitivity [152]. The most common forms are due to morphologic changes at the optic nerve head and cells of the retinal ganglion layer due to elevated intraocular pressure. Elevated pressure in the eye is often caused by poor drainage of fluid (aqueous humor) through the eye trabecular meshwork where the iris and cornea meet [137], but is also induced by chronic hypertension or buildup of plaque in blood vessels [153]. Progressive visual impairment is then defined by a chronic loss of retinal ganglion cells and their axons that comprise the optic nerve.

### 2.4. Opportunities for Microfluidics in Retinal Regeneration

Modern diagnoses of retinal disorders have been enriched by translational imaging modalities, biomimetic materials, and engineered tissue constructs. Microfluidics in regenerative medicine now enable singular opportunities for quantitative study of retinal cell responses to a range of therapeutic and clinically-applied stimuli. Forthcoming sections of this article describe both the microfluidic assays and microscale studies developed to elucidate fundamental cell behaviors and collective responses needed to restore vision.

## 3. Microfluidic Assays and Microscale Systems

**Figure 5:**
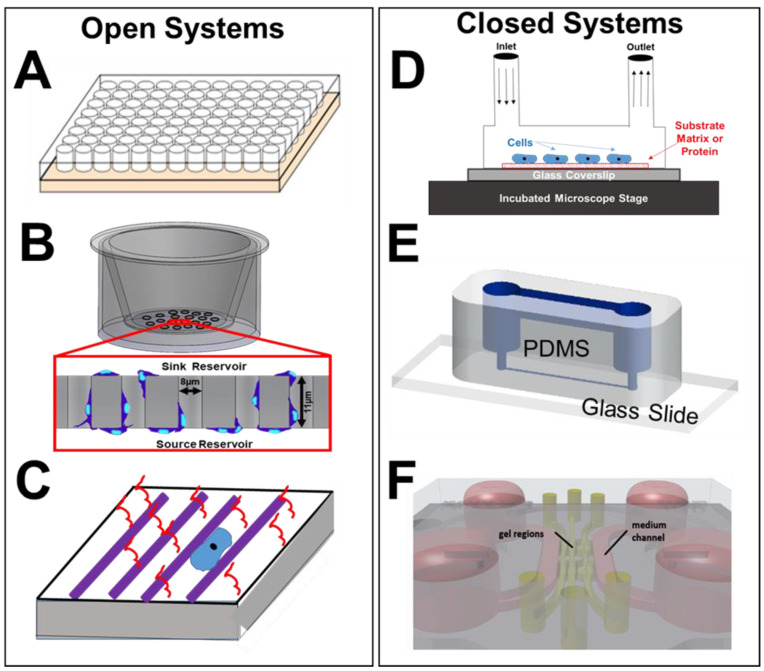
Description of prevalent microscale assays (open and closed) applied in biomedical studies and cell-based analyses of the retina. (**A**) A schematic of a multi-well substrate (hundreds to thousands of parallel wells) used for culture of individual cells or groups, as well as for study of cellular responses to extracellular solutions and matrix proteins. (**B**) Schematic of transwell assay (e.g., Boyden Chamber) where cells are inserted into a top, or sink, reservoir and migrate within the pores of a semi-permeable membrane towards the bottom, or source, reservoir. Motile cells then adhere to the underside of the membrane, are stained, and counted to estimate numbers of motile cells (reproduced with permission from [189]). (**C**) Rendering of a micro-patterned substrate, comprised of a flexible (or rigid) substrate that is functionalized with a densely-packed array of polymer substances in a pre-designed arrangement. Substances are additionally conjugated with proteins of interest for cellular interactions significant to guidance of cell processes or extensions, and cell culture when immersed in medium. (**D**) Schematic of a simplified micro-perfusion chamber, where cells are immobilized upon a coated glass side (or coverslip) and exposed to continuous flow via inlet and outlet ports. The chamber is placed within an incubated microscope stage for real-time observation of cell culture, growth, and alignment under flow conditions. (**E**) Schematic of microfluidic channel flow systems (reproduced with permission from [190]), where flexible substrates of PDMS or PMMA are bonded onto microscope slides (or coverslips) to control properties of the interstitial environment and facilitate visualization of cell behaviors. (**F**) Rendering of an on-a-chip device containing multiple, interconnecting layers of cells and flows to facilitate 3D modeling and organoid models, also known as micro-physiological systems. Two parallel culture chambers on the chip are separated by a central channel containing a pillared array of structures at both sides of the channel for interconnection (reproduced with permission from [186]).

A variety of in vitro assays have been developed or miniaturized in recent decades to examine the processes of intra- and extra-cellular signaling in retinal homeostasis and dysregulation, as well as to evaluate externally-applied stimuli to treat retinal disease [154]. Microfluidic and microscale technologies have manipulated (sub) microliter fluid volumes within micro-chambers and multi-well substrates, transwell assays and chemotaxis chambers, patterned substrates and microfluidic channels, as well as multiplexed devices and 3D cellular systems. Each microtechnology has enriched development of contemporary and emerging regenerative strategies through quantitative study of the individual and collective cell responses needed for vision. A summary of the most prevalent microfluidic assays is shown in Figure 5 at the end of this section.

### 3.1. Microscale Cell Culture Chambers

Retinal neurons and Müller glia have been examined, in large part, through in vitro cell culture, where surface-treated flasks or dishes are used to grow and observe cells outside of the body. Protocols of cell culture have been focal to the maintenance of primary retinal cells isolated from a wide range of animal models and clinical samples for over a half century [155]. In vitro culture is also credited with the development of immortalized retinal cell lines, which are central to in vitro study. These cell models possess useful characteristics, including contact inhibition, anchorage-dependent growth, expression of retinal-specific genes, and limited functional processes. Retinal progenitor cells have been modeled using the R28 cell line [156], which possess receptors that respond to neurotransmitter stimuli but lack voltage gated channels. Photoreceptors are often modeled using the 661W line [157], whose cells exhibit spindle-like processes and demonstrate biochemical characteristics exhibited by cones to aid study of photoreceptor-associated diseases [158]. The MU-PH1 Müller-derived cell line has also been recently applied to model photoreceptors because the cells are light sensitive and expresses opsin photoreceptor markers [159]. Interneurons are similarly modeled using cell lines, where behaviors of retinal horizontal cells have been represented by RB116 and WERI-RB27 models derived from retinoblastoma that express stem-like and retinal cell markers [160,161]. Responses of amacrine cells are modeled using the neuro retina cell line QNR/D [162], while behaviors of retinal ganglion have been heavily, and controversially, explored using commercial models of RGC-5 [163]. Moreover, immortalized cells of virtually all retinal neurons have been developed from SV40 T-antigen-induced tumors in transgenic mice [164]. While, immortalized lines often lack many functional characteristics of their in vivo response (e.g., photo-transduction or synapse), live cultured cells serve as important models for study of fundamental cellular processes and behaviors, and significantly aid proof-of-principle experiments for emerging biotechnologies.

The classic cell culture model is the static two-dimensional (2D) system developed nearly a century ago [165,166]. In this model cells are: (1) Immersed within milliliter volumes of culture medium solution, containing growth factors, glucose, and ions needed for cell survival; (2) allowed to adhere onto surface-treated substrates; and (3) left to proliferate until achieving near full coverage of the substrate surface, known as confluence. At this last stage, cells are detached from substrate surfaces, re-suspended in replenished medium, and re-plated onto different substrates to repeat the process as needed. This method has endured for its simplicity and ease of operation, but remains dependent on periodically replenished medium to avoid cell death caused by a lack of nutrients or oxygen and excess metabolic waste.

Commercial miniaturization of static 2D culture systems leveraged the lower costs and increased adaptability of microfabrication technologies to manipulate (sub) microliter volumes of specialized reagents. These new micro-culture systems permit study of single or multiple cell groups, in parallel, with the added benefit of minimized reagent quantities. Contemporary 2D cell culture is, thus, now readily performed in microchambers, where microfluidic volumes are enclosed directly upon a microscope slide to enable the growth of cell groups on the slide itself. Large scale parallelization of microchambers yielded multi-well and microtiter plates, where hundreds to thousands of (sub) microliter compartments, or wells, are fabricated in a uniform footprint on a single substrate [167]. It is noted that although the substrate length of conventional multi-well plates is often several millimeters long, the characteristic length of individual, parallelized wells is well within the μm range while the wells themselves encompass fluidic volumes of 1 μL or less. Experiments using multi-well compartments are able to monitor individual cells, or cell groups, to deliver minute concentrations of targeted reagents and pharmacology. These parallel microscale assays have significantly improved the resolution of 2D culture systems to examine retinal responses to extracellular compounds, including combinations of growth factors and cytokines, currently explored as key components of many regenerative therapies [105].

### 3.2. Transwell Assays

Compartments of microliter volumes have been widely-used to measure numbers of cells that migrate in response to extracellular signaling from one chamber to another. Traditional assays, such as Boyden or Dunn chambers [168,169,170], use transwell configurations to examine cell migration through fluidically-connected compartments either vertically (atop one another) or horizontally (side by side). Migration assays are typically comprised of two volumetric reservoirs separated by a permeable membrane with micrometer diameter pores. Live cells are inserted into one compartment and stimulated to migrate through the porous membrane towards the opposite, reagent-filled compartment. Stimulated cells migrate in response to chemical concentration gradients, a process called chemotaxis, to undergo full body translocation and penetrate the pores of the membrane. These motile cells remain adhered to the membrane underside and are then stained and counted to represent the migration strength, or chemotactic potential, of the reagent tested. NS projects have used various metrics to represent chemotactic potential, including the raw numbers of motile cells counted compared to control solutions (buffer or media), the migration fold increase in motile cells over control, or the relative chemoattractant factor [171,172], i.e. a normalized parameterdefined as the cell count per reagent divided by the cell count per control solution. Transwell assays have provided significant contributions to regenerative medicine by identifying chemo-attractive molecules able to stimulate in vitro motility of retinal cells and their progenitors, as well as by examining the chemo-attractive strength of molecules expressed, in vivo, at various stages of degeneration or pathology [173].

### 3.3. Micropatterned Substrates

Microfabrication processes developed for other areas of engineering, such as semi-conductor and electronics, have notably enhanced the in vitro study of cells via surface micro-patterning, i.e., the processes by which precisely-textured arrays of targeted biomolecules are created atop cellular substrates. Micro-patterned systems have been increasingly used to examine surface effects on localized cell response in biotechnology and regenerative medicine. Patterns are produced using (sub) micrometer features that are deposited or etched onto rigid or flexible substrates, such as silicon or elastomers, and functionalized with a variety of extracellular proteins and matrixes. These substrates are developed to stimulate changes in cell responses to a range of physiochemical stimuli significant to NS function, such as substrate rigidity and adhesion sites [174]. Micro-patterned assays, thereby, provide new tools with which to explore the behaviors of anchorage-dependent cells within their localized microenvironment, where topographical cues may influence cell response or function. The assays have been significant to regenerative medicine because signaling pathways known to stimulate and/or regulate the fate of stem-like cells can be targeted via localized cell-surface interactions [175], including the distribution and density of binding proteins [176], cell-surface contact area, and mechanical compliance of adhesion substrates.

### 3.4. Microfluidic Channels

Micro-manufacturing from computer chip applications have been similarly applied to produce microfluidic channels with anatomical dimensions and geometries on the micrometer scale of biological systems. These microfluidic devices are manufactured using conventional photolithography on wafer substrates to create durable and reproducible molds, or through so-called soft lithography, using polymeric substrates to create more pliable and cost effective elastomers that interface with biological components, such as cells and tissues [23]. In both cases, the substrate containing the microfabricated design is chemically bonded to another substrate, such as microscope slide or coverslip, to create enclosed microfluidic environments that enable survival and growth of biological specimens [177]. Microfluidic channels create defined microenvironments for cell culture and stimulus by using combinations of convective flow, diffusion, electrokinetic fields, and other phenomena to regulate extracellular properties, such as chemical concentration, ionic strength, or substrate adhesion [16,178]. These microsystems are then able to correlate measured cellular changes with the defined extracellular stimuli imposed. As an added benefit, microfluidic flows can continuously monitor and distribute nutrients and oxygen within closed microenvironments and remove toxic waste products of cell metabolism, simultaneously. These advantages have made microfluidic channels fundamental tools with which to examine a breadth of cell processes significant to retinal regeneration, including individual behaviors of adhesion and morphology, as well as single cell chemotaxis and collective migration, cell to cell connectivity and synapse, among many others [179,180,181,182].

### 3.5. Microfluidic Perfusion Chambers

Projects in regenerative medicine have begun to apply long-term culture within microscale perfusion chambers to isolate and characterize potential replacement cells [41]. Perfusion chambers are assays comprised of one or more cell culture compartments connected to a hydraulic system that facilitates rapid fluidic renewal. Perfusion chambers apply flow conditions during the culture of cells upon different extracellular matrixes or within bio-scaffolds. Their continuous supply of medium from periodic volume exchange prevents waste accumulation, while simultaneously permitting cells to create a stable environment under flow [183]. Microperfusion projects have characterized and measured concentrations of proteins secreted by a variety of cultured cells, where recent contributions have elucidated the chemical responses of potential stem-like cell replacements to external cues.

### 3.6. On-a-Chip Devices

Microfluidic channels have been parallelized and interconnected to facilitate increased complexity in the in vitro modeling of tissues [184]. On-a-chip technologies are integrative, microfabricated platforms designed to recapitulate functional units of tissues in vitro [185,186]. Novel micro-physiological systems (MPS) are 3D cellular models where cells grown within extracellular matrix reflect critical aspects of tissue function, including cellular interfaces and responses to physiochemical stimuli from flow and pressure. Fluids within MPS are restrained in microfluidic channels, enabling close contact between different cell types to capture dynamic cell-cell interactions, while properties of the microenvironment critical to mimic tissue function are examined in the same setup, e.g., spatiotemporal chemical gradients [187] and mechanical strain [188]. The optical transparency of MPS similarly facilitates real-time monitoring of cellular processes significant to the analyses of tissue functions during development and regeneration.

Microfluidic devices and in vitro assays have been indispensable in the development and advancement of regenerative medicine in the retina. Microtechnology has enabled ground breaking opportunities for the study and manipulation of stem cells, and become equally transformative in the study of stem cell responses to adult retinal cues needed for regeneration.

## 4. Modeling of Retinal Cell Behaviors

**Figure 6:**
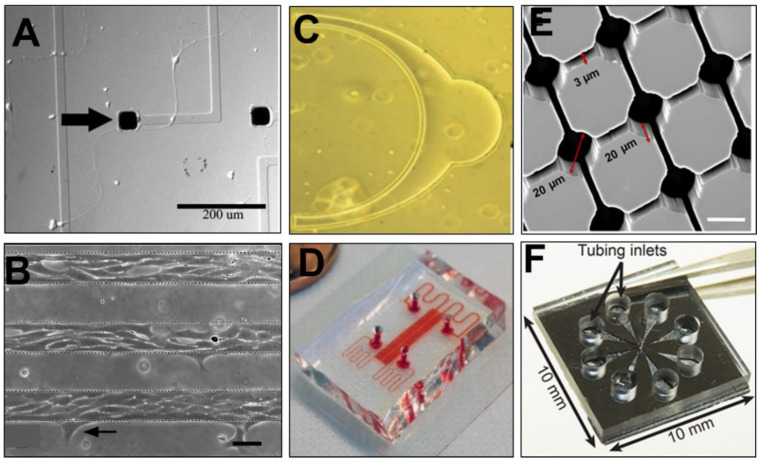
Selection of contemporary, microfluidic and microscale assays developed for bioengineering study of the visual system. (**A**) A micropatterned substrate illustrating the growth and guidance of retinal ganglion axons towards electrodes with and without substrate protein conjugation (image reproduced with permission from [249]). (**B**) Retinal cells growing preferentially on a poly (dimethyl) siloxane (PDMS) substrate whose surface is functionalized with patterned layers of polyelectrolyte multilayers (image reproduced with permission from [59]). (**C**) A microfluidic anatomical model, called the μ-Retina, designed to produce concentration gradients of chemoattractants for retinal stem-like cells using in vivo scale and spherical geometries (image reproduced with permission from [219]). (**D**) A microfluidic device, called the Gal-Mμs system, which employs microfluidic channels, nanoarrays, and electrical stimulation to stimulate motile and adhesive behaviors of photoreceptor precursors (image reproduced with permission from [223]). (**E**) A neural network chip, called the NN Chip, comprised of interconnected microwells to examine photoreceptor connectivity and apoptosis (image reproduced with permission from [244]). (**F**) A microfluidics-based, synapse chip used for neurotransmitter modulation of explanted retina (image reproduced with permission from [248]).

Multifaceted cell behaviors are fundamental to the development and growth of retinal tissue, as well as to its pathology, disease progression, and response to insult and injury. Regenerative medicine seeks to restore vision, in part, by recapitulating endogenous cell processes essential for retinal function. These include behaviors, such as survival and death, adhesion and migration, as well as more specialized processes of intercellular connectivity and synapse. Descriptions of select microfluidic and microscale assays are shown in Figure 6 at the end of this section.

### 4.1. Survival and Viability

Survival of retinal cells outside of the body is critical to the in vitro methodologies on which development of many regenerative strategies rely. The long-term viability of replacement cells in vivo is essential because it facilitates the synaptic integration desired to restore vision. However, these replacement cells must survive isolation, characterization, and manipulation in vitro prior to in vivo application [13]. Numerous studies have utilized micro-wells and micro-chambers to culture retinal cells and extract and collect their extracellular medium or supernatant. The so-called enriched, or conditioned, medium has been applied to increase survival of primary retinal cells and promote viability of retinal cells isolated from animal models or clinical samples. While, applications of enriched media is well-established across the NS [191,192], its molecular screening for retinal compounds has only recently become feasible through newfound interfaces with microfluidic assays. Parallelized multi-well plates now facilitate biochemical analyses using (sub) microliter volumes of extracellular fluid, whereas the requirement of milliliter volumes limited large scale analyses in retinal applications just a decade ago. Microliter to nanoliter volumes of extracellular fluid collected in parallelized wells readily flow in low Reynolds number regimes, whose highly parallel streamlines facilitate diffusive transport of picomolar to femtomolar concentrations of extracellular compounds. Applications of micro-culture analyses have further examined the influence of dilute concentration fields of such targeted external factors to report higher viability of primary photoreceptors cultures when cultured with fibroblast growth factor (FGF), epidermal growth factor (EGF) and insulin-like growth factor-binding protein-5 (IGFBP5) [193,194]. Photoreceptor viability was additionally increased when cultured with conditioned media containing connective tissue growth factor (CTGF) [195], while conditioned media with pigment epithelium-derived factor (PEDF) promoted in vitro survival of retinal ganglion cells [196]. Multi-well plates have similarly facilitated large scale screening of retinal responses to emerging pharmacology, where primary photoreceptor cultures were used to evaluate the neuroprotective effects of the anti-inflammatory drug minocycline [197] and micro-cultured retinal ganglion cells used to examine the toxicity of the anti-VEGF drug, Avastin [198].

### 4.2. Morphology and Cell Growth

In vitro cell morphology has become increasingly significant to regenerative medicine, as cell shape is believed to portend intrinsic determinants of endogenous cell growth and repair processes [11,199]. Morphology varies greatly across different subtypes of retinal neurons and glia, and has been measured using optical microscopy in tandem with a number of microfluidic and microscale substrates. The endogenous shape, or morphology, of a retinal cell is an important indicator of both its health and function in the visual system, as first described by the pioneering neuroscientist Ramón y Cajal [200]. Cell shape has been assessed using a variety of quantitative parameters [176,201,202], including average numbers of branched and unbranched cellular extensions, average diameter of cell bodies, maximum end-to-end lengths, as well as cell shape index, a dimensionless parameter that relates cell surface area to its perimeter.

In vitro, microscale study of amacrine cells have provided an excellent model system with which to study intrinsic determinants of cell growth because of amacrine morphologic heterogeneity. Microscale systems, such as micro-wells and microstructures upon optical substrates, have greatly enhanced the ease of cellular imaging at increasing resolution to now characterize cell morphology using multiple parameters on the micro and nanoscale. Amacrine cells exhibit a widely varied and complex neurite patterning, with over 30 morphologic subtypes that include few to multiple neurites, short or long axon-like processes, and thin or bushy dendritic-like trees [203]. It is believed that these changes in morphology are related to different mechanisms of synaptic transmission between amacrine cells and retinal ganglion cells needed to effectively transmit photonic signals to the optic nerve. A recent study used miniaturized micro-well plates to culture retinas of Sprague-Dawley rats dissected from embryonic day 20 (E20) and postnatal days eight through 10 (P8–P10) [203]. Experiments in the microfluidic environment reported that a significant portion of amacrine cells extended neurites over 150-um-long, consistent with in vivo data showing post-natal amacrine cells, able to extend multiple neurites of different average lengths. The in vitro data suggested that amacrine cells have cell subtype-specific intrinsic determinants of neurite growth that are carried into culture. This behavior would significantly aid communication with host neurons during transplantation therapy and has guided many regenerative projects to begin selection and characterization of stem cells for intrinsic morphological determinants of endogenous growth [76].

Rod bipolar cells are similarly significant to retinal regeneration because they connect directly to photoreceptors to aid transmission of photonic signals [204]. Microscale cultures were utilized to examine properties of rod bipolar neurite outgrowth in an early study [205]. Rod bipolar cells were grown in short term culture as isolated cells within microfluidic chambers, as well as within microfluidic volumes of retinal explants. Both sets of data reported extended dendritic processes that were decorated with varicosities and smaller spine-like extensions. This in vitro contribution was striking because the morphology of rod bipolar cells observed had not been reported in healthy retina, and furthermore resembled that of bipolar cell remodeling observed in models of retinal detachment. While, it remains unclear whether these dendrites possess the synaptic machinery to create functional synapses, latent ability of rod bipolar cells to extend different morphological processes in vitro might enable its integration into degenerated retina, or likewise promote connections with transplanted cells.

Retinal ganglion cells have been extensively studied in regenerative medicine because of their direct relationship with the optic nerve. Mammalian retinal ganglion cells consist of over a dozen subtypes, each clearly distinguishable from one another in structure and function. A recent study used micro-patterning to direct neuronal growth of ganglion cells for single cell stimulation [206]. Experiments used buffered solutions to direct the growth of retinal ganglion cells isolated from Sprague-Dawley rats to individual electrodes of a planar microelectrode array. Varying concentrations of neurotransmitters were then used to achieve selective cell stimulation at different thresholds. A complementing study developed a so-called synapse chip [207], which used micro-patterned substrates and microfluidic channels to direct individual ganglion neurite growth by delivering localized neurotransmitters. These results highlight advantages of microfluidic technology to apply stimuli that direct the growth of cellular neurites needed for adult regeneration. Notably, retinal ganglion cells have been the primary targets of retinal prostheses, whose micro-machined implants stimulate cells directly using electrical signals. Reviews of the advancement and clinical translation of these implantable MEMS systems can be found in the following recent articles [208,209].

Micro-fabricated structures have also been used to examine the shape or size of retinal stem cells isolated for cell replacement therapy. A recent study [210] developed a micro-device using photolithography that promoted individual cell tumbling of retinal stem cells for in vitro selection. This project developed a microscale device that used notched substrates for determinate lateral displacement, well-known to promote cell sorting. The device utilized offset arrays of posts with designated periodicity between rows to sort cells groups above critical diameters. This microsystem sorted cells using 5 distinct size-based populations, where notched substrates enhanced distinction between single cells compared to conventional circular posts. The project illustrated that single-cell tumbling enabled high-resolution molecular profiling of retinal stem cells based on individual size. Data further suggested that retinal cells of given morphology, or size, may be derived from specific sub-populations of endogenous stem cells. These contributions have significantly enriched the microfluidic sorting of transplantable stem cells targeting specialized cell groups for cell replacement, such as rods and cones [113].

### 4.3. Migration and Modalities

Many cell replacement therapies rely on the localized migration of transplanted retinal progenitor cells within host tissue for synaptic integration. Importantly, recent landmark studies illustrating material transfer from transplanted progenitors to models of genetic retinal disease have, thus far, reported the absence of this phenomena in transplantation to degenerated, adult retina [211]. While, terminally-differentiated neurons of the mammalian retina are unreported to migrate in vivo, the migration of retinal progenitors has been extensively studied in developmental biology [212]. Cells can migrate in response to changes in substrate surface molecules and stiffness via haptotaxis, as well as towards gradients of biochemical factors or changes in electric potential via chemo-, and galvano-taxis, respectively. Recent biomedical engineering studies have developed microfluidic and microscale assays to examine the in vitro migratory responses of transplantable retinal progenitor cells toward a variety of external stimuli significant to adult tissue.

Retinal progenitor migration was examined in early ophthalmic studies via microscale diode lasers during photocoagulation, i.e., processes that use heat to shrink or destroy abnormal retinal structures. One project reported that laser injury promoted migration and integration of retinal progenitor cells into host retina [213], which provided early enthusiasm for altering the extracellular matrix (ECM) during transplantation. A newer study examined the ability of transplantable biomaterials to induce the migration of retinal progenitor cells using microfluidic channels [214]. Here, channels were fabricated via elastomeric molding and bonded onto microscope coverslips to create closed systems of characteristic length ~150 μm, i.e., the approximate distance between cell insertion into the sub-retinal space and the photoreceptor layer. The project examined the collective adhesion and displacement of retinal progenitor cells upon substrates of poly-l-lysine, fibronectin, laminin, hyaluronic acid, and Matrigel to show different adhesion patterns upon each polymer surface. Tests further applied a chemical gradient along functionalized surfaces to examine progenitor chemotaxis. The results illustrated that constituent proteins of transplantable materials promoted individual or collective cell migratory behaviors to the same stimulus. Numerous biomaterials have since been developed to examine substrate-induced migration by using polymeric compounds of proteins endogenous to the inter-photoreceptor matrix and retinal extracellular matrix, such as hyaluronic acid, collagen, chondroitin sulfate, and laminin. While, emerging transplantable biomaterials have generated excitement to bolster outcomes of cell replacement therapy, their tissue engineering methodologies rely largely upon chemical interactions rather than microfluidics with the notable exceptions of microfabricated scaffolds. The reader is, here, directed to current reviews of emerging retinal biomaterials [215,216].

Retinal cell migration has been most recently measured using chemotaxis and transwell chambers produced via microfluidics. These contemporary systems permit the study of cell morphology changes during substrate adhesion and motility, as well as signaling pathways activated in response to chemoattractive gradients. One study developed microfluidic transwell inserts that generated tissue culture-friendly gradients within multi-well plates [217]. The self-supported device is hung above the cell culture surface to create gradients via small microflows across a track-etched porous membrane. This microassay delivers stable and measurable gradients over a large area with minimal shear stress to dissociated cells or to tissue explants cultured independently on the surface of the multiwell plate. The device was applied to a retinal explant isolated from an E15 embryonic mouse to show that external fields of brain-derived neurotrophic factor (BDNF), cytokine ciliary neurotrophic factor (CNTF), and second messenger gradients (cAMP) stimulated cell outgrowth in the preferential direction of gradients. These chemoattractive factors, among others, have been further examined using microfluidic chemotaxis chambers, which employ microfluidic channel(s) to create precise chemical concentration gradients that enable concurrent real-time cell visualization.

Precise manipulation of the cell microenvironment within local segments of microfluidic channels facilitates measurement of different parameters to evaluate cell migratory behaviors, such as directionality of motion, cell polarization, path length, and net distance traveled. Moreover, these metrics are readily assessed by plotting real time cell trajectories against concentration gradients within the channel to facilitate more comprehensive study of cell migration than the cell numbers provided by transwell assays alone. A recent microfluidic chemotaxis chamber was developed to evaluate progenitor chemotaxis in response to a panel of extracellular growth factors [218]. This system, known as the μ-Lane, used diffusive transport to evaluate transient and steady-state concentration profiles of epidermal growth factor (EGF). The results illustrated that retinal progenitors were chemoattracted by different concentrations of EGF and further linked this migratory behavior to intracellular JAK/STAT and PI3kinase signaling pathways. However, measurements of cell path length and net distanced traveled indicated that progenitor migration towards EGF was non-directional, or chemokinetic, meaning EGF fields alone would be unable to direct retinal progenitor movement along trajectories or towards desired targets. An additional study by the same group integrated convective-diffusion mechanisms within the device to create non-linear concentration gradients. The results determined that transplanted cells exhibited strong chemotactic behavior towards concentration gradients of platelet-derived growth factor (PDGF), vascular endothelial growth factor (VEGF), glial cell-derived neurotrophic factor (GDNF), and stromal-derived growth factor 1 (SDF-1). Moreover, retinal progenitors migrated towards SDF-1 along large migration distances and in the direction of steeper concentration gradients than the other factors examined. These contributions were among the first to suggest that non-linear, extracellular chemotactic fields can be used to direct the migration of transplanted cells into retina, which remains a principle challenge in cell replacement therapy of adult degenerated retina.

A microfluidic model of mammalian retina was produced using anatomical geometry and micrometer dimensions reported from mouse and human [219], as shown in Figure 6. These researchers used soft lithography to develop the μ-Retina, a novel and biomimetic device able to examine cell migratory behaviors within spherical retinal geometries produced at the in vivo scale. The results established SDF-1 as a potent chemoattractant of transplantable retinal progenitors, as the cells were observed to migrate acutely towards a narrow range of SDF-1 concentration gradients created using different ECM substrates in both 2D and 3D. This project additionally illustrated the ability of contemporary microfluidics to examine both individual and collective cell migratory behaviors. Real-time visualization illustrated that extracellular gradients stimulated retinal progenitor cells to self-organize into collective cell groups, or clusters, for more efficient collective migration [220]. Data showed that extracellular SDF-1 fields stimulated formation of retinal progenitor clusters, but not of other cells derived from the NS, such as oligodendrocyte progenitors. Further, expression of the SDF-1 receptor, CXCR4, and the gap junction protein Connexin 43 (Cx43) revealed that only clusters of retinal progenitor cells with elevated Cx43 expression exhibited collective migration along SDF-1 concentration gradients. These contributions highlighted the significance of collective cell responses rather than the behavior of individual cells, alone, which has guided many emerging regenerative transplantation studies [143].

Follow-up studies from this group then adapted a micro-nanofluidic system [221,222], called the MμN system, to examine the effects of electric fields on retinal progenitor migration. This system was comprised of two distinct microliter cell reservoirs connected by an array of nanometer-sized channels, and fabricated using a two-step photolithography process. The concentration gradients across the nanoarray were developed from one reservoir to the other, while two columns of agar with embedded platinum wire were used as electrodes across the cell reservoirs. The results illustrated that electric fields stimulated slightly larger migration distances than chemical fields, but with approximately the same directionality. However, stimulation from concurrent electric and chemical fields of SDF-1 resulted in five-fold increases in progenitor migratory distances and directionality of movement towards increasing gradients in the device [223], as per Figure 6. These novel results provided the first indication that combined fields can direct retinal progenitor migration very precisely, and have led the group to examine the combined use of these therapeutic fields in ex vivo models of inoculated eye [224].

Newer microfluidic studies have begun to examine the migratory roles of Müller glia in retinopathies driven by chronic metabolic syndromes, such as adult diabetes. As microfluidic transwell assays have illustrated chemotactic behaviors of Müller glia towards endothelial-associated factors, such as transforming growth factor beta (TGF-b) and fibroblast growth factor (FGF) [225,226] a recent microfluidic project developed a chemotaxis chamber called the glia line, or gLL, to more quantitatively evaluate the chemotaxis of cultured models of Müller glia [227]. Results validated preferential glial migration towards concentration gradients of EGF, VEGF, and FGF, previously reported in the literature. Experiments further used a large range of concentration gradient fields and commercial inhibitors to highlight mechanistic signaling from the MAPK pathway in the chemotaxis of Müller glia towards EGF [190]. Recent work from this same group has examined the interplay of the EGF receptor and VEGF ligand within gradient fields of EGF in microfluidic environments [189]. Surprisingly, the results demonstrated that VEGF upregulated EGF-R expression nearly 10-fold more than the cognate EGF ligand to increase Müller glia chemotaxis. This contribution is highly significant to the development of pharmacology treatments for vascular-related retinal eye diseases, such as anti-VEGF compounds (Avastin), growing in usage among adults worldwide.

### 4.4. Apoptosis and Cell Death

Apoptosis is the genetically-regulated process of cell death in multicellular organisms. Programmed cell death is central to the pathology of many retinal diseases and critical to development during retinogenesis. In adult tissue, apoptosis propagates biochemical stimuli that dysregulate retinal homeostasis and accelerate degeneration via cell to cell connectivity [228]. A recent microfluidic study of the causes and propagation of localized cell death signals has aided development of regenerative treatment of widespread retinal disease.

The apoptosis of primary rod and cone neurons via chronic light exposure has become heavily investigated in response to our hyper-exposure to illuminated screens via smart phones, tablets, and laptops [229]. The retina is particularly prone to oxidative damage because of its high oxygen consumption and constant exposure to light. Excessive light exposure stimulates photo-oxidative mechanisms that lead to significant cell death of photoreceptors, amacrine cells, and bipolar cells. A microfluidic perfusion chamber was recently used to examine apoptotic signals of retinal neurons in the inner nuclear layer [230]. Mouse neuro retinas were peeled from the RPE, incubated in a chamber, and treated with dual linear flow (interface) of artificial cerebral spinal fluid. The study modeled ischemia, i.e., reduced blood flow and oxygen, to illustrate that rod bipolar cells and amacrine cells released measurable glutamate in vitro during retinal insult and death. Excessive glutamate increased both intracellular calcium (Ca^2+^) and nitric oxide (NO) production through activation of the N-methyl-D-aspartate (NMDA)-type glutamate receptor, resulting in the apoptosis of these secondary retinal neurons. A separate study used multiwell plates to illustrate that chronic light exposure to short wavelength, blue light (450–485 nm) can cause cell death in photoreceptor models via elevated mitochondrial metabolism [231]. Microfluidic well plates were further used to examine the formation of reactive oxygen species in retinal tissue, which is believed to contribute to neurotoxicity in acute and chronic neurodegenerative diseases. The project used photoreceptor cells in an ischemia-reperfusion (I/R) model [232] to examine neuroprotective roles of various factors, such as nuclear factor erythroid relative-factor (Nrf2), a key regulator of cellular anti-oxidant response. The pathogenesis of cellular injury in ischemia-reperfusion (I/R) is believed to include the generation of reactive oxygen species, which generates inflammatory processes to damage retinal cells. Experiments of this work used a potent Nrf2 activator on cultured photoreceptor cells under oxidative stress conditions to inhibit formation of reactive oxygen species and reported increased neuronal cell survival after I/R injury. These findings indicated that Nrf2 exhibits a retinal neuroprotective function in I/R to suggest that pharmacologic activation of Nrf2 could be a therapeutic regenerative strategy.

Apoptosis of retinal ganglion cells from chronic degeneration has also been examined using multi-well plates. One study demonstrated that short wavelength blue light negatively affected mitochondrial function in retinal ganglion cells to decrease survival. Interestingly, subsequent exposure to long wavelength, red light enhanced mitochondrial function to increase survival of cultured retinal ganglion cells [233] and reduce the effects of blue light. Cell death was similarly reported using photoreceptor cell models within microfluidic transwell assays, exposing cells to oxidized phospholipids and hydrogen peroxide that mimicked oxidative damage [234,235]. The results of these studies illustrated that in vitro apoptosis of primary and secondary retinal neurons can be used to mimic specific aspects of in vivo degeneration to aid the search for neuroprotective compounds and targeted pharmacology.

Microfluidic systems have additionally examined the apoptosis of retinal ganglion cells due to chronically elevated intraocular pressure, a hallmark of glaucoma [173]. As hydrostatic pressures have been utilized to model retinal ganglion apoptotic cell death in response to oxidative stress, one project increased volumetric depth within microfluidic wells to stimulate elevated intraocular pressure [236]. The results identified significant roles of the nerve growth factor (NGF) signaling pathway in cell death, acting via both protein kinase B- (AKT) and apoptosis signal-regulating kinase 1 (ASK1). A similar study used a custom micro-pump system to apply a range of hydrostatic pressures upon culture cells [237]. Data illustrated that elevated pressure caused mechanical compression of ganglion axons to affect transport of nutrients needed for survival. Microfluidic testing further demonstrated that the antioxidant activity of compounds such as Alpha-lipoic acid (ALA) promoted viability of retinal ganglion cells via abilities to scavenge reactive oxygen species, chelate transition metal ions, and enhance the antioxidant potency of other compounds. Lastly, a separate study used microfluidic, transwell co-cultures to examine the influence of localized stem cells on the apoptosis of retinal ganglion cells. The project [238] modeled oxidative damage via hydrogen peroxide (H2O2)-induced injury and co-cultured ganglion cells with bone mesenchymal stem cells (BMSCs). The tests measured decreases in the apoptosis of retinal ganglion cells that were correlated to the pro-inflammatory cytokines secreted by BMSCs. Data additionally illustrated that the co-culture environment promoted ganglion cell neurotrophic expression. These collective contributions have enriched regenerative strategies in adult retina by demonstrating that microenvironments can be regulated to increase their neuroprotective effects on retinal neurons [4].

### 4.5. Neuronal Connectivity

Vision requires extraordinary coordinated responses among retinal neurons and glia to produce images of objects in the visual cortex. Replacement cells must, therefore, possess specialized capabilities to establish cellular interconnectivity within existing retinal circuitry to restore vision. A variety of micropatterned substrates have examined cell to cell connectivity between primary neurons, interneurons, and retinal ganglion. A recent project [59] developed surface patterns that positioned cells (sub) micrometer distances apart, as per in vivo spacing, and used microfluidic networks to examine connectivity between retinal neurons on functionalized surfaces. Micropatterned polyelectrolyte multilayer (PEM) lines were adsorbed onto poly (dimethyl) siloxane (PDMS) surfaces and were connected through adjoining microchannels, as shown in Figure 6. Retinal cells were adhered onto PEM lines and elongated within microchannels to achieve connectivity with neighboring cells. Changes in cell to cell connectivity were further observed upon PEM surfaces coated with a variety of polymer matrixes, including poly (ethylenimine) and poly (allylamine hydrochloride) that possessed positively-charged amine groups. These contributions have greatly aided the development of biomaterials seeking to promote synaptogenesis between transplanted cells and host neurons. As a result, several bio-scaffolds have emerged that produce lattices of retinal matrix proteins to support retinal cell viability and interconnectivity. While, the wealth of emerging retinal biomaterials remains outside the scope of the current article, select contemporary articles can be found here [215,216].

Micropatterned systems have also investigated how synaptic communication with retinal stem cells is influenced by the regional cell origin [239]. Regional differences in cell type densities and resulting positional cues suggest that retinal topography may contribute to the lack of integration reported when using transplanted stem cells. In vivo evidence for retino-topography is well-established in the outer nuclear layer, where photoreceptors depend upon their surroundings for proper differentiation [240,241] while their direct communication with bipolar cells is unevenly distributed with greater densities in different retinal layers [242]. Moreover, different distributions of differentiation factors across the retinal surface have direct effects upon photoreceptors during development [243]. One project micropatterned the surface of microfluidic channels with antibodies to create a pattern where each photoreceptor had only 1–2 neighboring cells along the microchannel longitudinal axis. This patterning along single-cell lanes enabled study of interactions between cell pairs separated from other cell-cell neighbors. Experiments using this microfluidic system revealed new specificities in the connectivity preferences of photoreceptors to suggest unexplored retinal topographical preferences that may significantly impact cell replacement therapies.

Microfluidic channels have similarly examined the cell-cell interactions needed for synapse formation between retinal neurons. One study developed a neural network chip, called the NN-Chip, as per Figure 6, to examine synapse formation between photoreceptors and progenitor cells [244]. The device was comprised of ordered microwells connected by microfluidic channels on a square lattice. Tests loaded individual cone photoreceptors into microwells and allowed the cells to connect and form synapses within microchannels to build a neural network. The NN chip illustrated that synapse across retinal neurons occurred via gap junctions, i.e., specialized channels between the cytoplasm of connecting cells that allow transmission of ions and electrical impulses [245]. While gap junction regulation of retinal synapse has been well-established, this project examined its role in cell death via the so-called bystander effect, a phenomena where dying cells cause deterioration of adjacent cells to propagate neurodegeneration [246]. The bystander effect in retina impairs neuronal connectivity and impulse synchronization to cause progressive and irreversible vision loss. By observing the distribution of apoptosis that spread from light-induced apoptotic cones to surrounding cones, the group was the first to model in vitro, bystander apoptosis in retina. The project data suggests that gap junctions formed via connexin 36 (Cx36)-containing channels acted as tunnels to enable passage of apoptotic signals between cones throughout the entire photoreceptor layer. These contributions have greatly aided contemporary understanding of retinal dependence on interconnectivity for apoptosis to aid development of regenerative strategies to treat degeneration.

A different project examined retinal synapse across retinal progenitor cells cultured within a multi-channel, microfluidic chip [247]. Glycinergic factors were added onto cultured retinal cells physically connected through microchannels to illustrate their electrical activation in vitro. Another recently prototyped synapse chip [248] examined neurotransmitter stimulation of explanted retina using an array of independent microports connected to on-chip glutamate reservoirs via microchannels. Experiments using this multiport, microfluidic device, shown in Figure 6, were the first to examine the in vitro levels of glutamate neurotransmitter to illustrate spatial patterns of retinal stimulation. The artificial synapse chip was further used to achieve localized chemical release via electro-osmotic delivery of chemical compounds at multiple chip surface locations [249]. These contributions have elucidated in vitro synapse functions as well as utilized microfluidic intervention to stimulate regeneration.

### 4.6. Neuronal-Glia Communication

Retinal glia are mechanosensors that maintain homeostasis as well as initiate neuroprotective responses to retinal insults. Müller glia are well known to ensheath retinal vasculature, provide nutrients and neurotransmitters, and act as light scattering cells for enhanced visual acuity [146,189]. However, their direct communication with the different groups of retinal neurons remains incompletely understood. Several contemporary microfluidic studies have recently examined communication between retinal neurons and Müller glia to aid development of cell-based regenerative therapies.

One study used microfluidic culture wells to examine the significance of interactions between Müller glia-retinal ganglion cells as a function of lactate [250]. The retina is a highly metabolic organ having up to 10-fold higher concentrations of lactate compared to other body tissues and blood. The project determined that Müller glia increased lactate metabolism to elevate ATP production in ganglion cells. A separate study used microfluidic wells to demonstrate significant increases in the survival of retinal ganglion cells when cultured with Müller glia [251]. This project illustrated that glia were able to increase ganglion cell survival by via uptake of glutamate. Importantly, glia were further seen to increase ganglion cell survival after mitochondrial inhibition, demonstrating their significant roles in the regulation of retinal metabolism. These contributions highlight the importance of interactions between Müller glia and retinal ganglion in retina survival, which are currently explored as part of emerging therapies [106].

Microscale technologies were additionally applied to examine behaviors of Müller glia significant to the neuritogenesis of retinal ganglion cells. In one study [252], Müller glia were grown to confluence on a micro-well membrane with retinal ganglion cells co-cultured on top. The results illustrated that the portion of ganglion cells expressing neurites increased more than two-fold when grown atop glia. In addition, neurite lengths increased two-fold to for extensions 50 to 200 μm in length and numbers of retinal ganglion cells with neurites more than 200 μm in length increased four-fold. These results illustrate that Müller glia aid ganglion neurite extension, which is known to significantly promote synaptogenesis with transplanted cells.

A separate study used microdevices to investigate the specific reaction of Müller glia and neurons to oxidative damage induced by hydrogen peroxide (H_2_O_2_) injury [253]. Via separate microfluidic co-cultures of Müller glia and photoreceptors or amacrine cells, this project illustrated that glia in vitro reacted to injury by rapidly activating different pathways that supported retinal function. A similar project established an ex-vivo, co-microfluidic culture system using primary retinal ganglion cells and Müller cells to examine levels of the excitatory amino acid homocysteine (Hcy), implicated in reactive ion species [254]. The study reported that while Hcy-exposure decreased viability of retinal ganglion cells, Müller glia produced a robust mitochondrial and glycolytic response. Moreover, findings demonstrated that Müller glia are first responders to oxidative stress and are able to de-differentiate and re-enter the cell cycle to replace damaged cells. These contributions have highlighted Müller glia as new targets for regenerative medicine by de/differentiation into progenitors used in transplantation. Recent projects have heavily examined Müller glia for processes of stem cell renewal and for gene therapy, as discussed in several excellent articles [255,256,257].

Microscale technologies have further examined cellular process and connectivity affected by changes in blood glucose levels. Elevated glucose concentrations can lead to the onset of adult diabetic retinopathies, which have been rising steadily worldwide and sharply in the United States [89]. One study [258] used micro-well plates to examine the response of retinal ganglion cells and MG when exposed to Dexamethasone (DEX), a corticosteroid used for its anti-inflammatory and immunosuppressant effects. Retinal ganglion cells and Müller glia were isolated from adult female Sprague-Dawley rats and the cells were seeded into micro-wells both individually and in co-culture. Tests illustrated that high concentrations of glucose decreased the survival of retinal ganglion cells when cultured alone or with Müller glia. However, DEX prevented ganglion cell death in hyperglycemic conditions, at least partially by dampening the release of its cytokines. The anti-inflammatory properties of DEX rescued ganglion cells from death to suggest that inflammation plays a critical role in the glucose-induced ganglion death in vitro. Complementing studies then examined the effects of glucose deprivation using micro-well plates and illustrated increased oxidative stress in photoreceptors [259]. The results demonstrated that autophagy, i.e., mechanism to remove dysfunctional cell components, was significant for photoreceptor survival under hyperglycemic conditions [260]. It is noted that, while several microsystems have been developed to model diabetic retinopathy, these projects surprisingly neglect Müller glia and focus almost exclusively on endothelial cells [261,262,263]. Future microfluidic systems are needed to develop in vitro models of diabetic retinopathy and blood retinal barriers to advance regenerative treatments for vascular and metabolic eye diseases.

Contemporary microfluidics have evolved into lab-on-a-chip devices, where whole or tissue segments can be examined in a microfluidically-controlled environment. A recent retina-on-a-chip device was developed to facilitate the culture, manipulation, and probing of explanted tissue [264]. The design is composed of twelve access channels and a single suction channel with characteristic dimensions of hundreds of microns. The suction channel used to pull the retina into contact with the through-holes formed an X-shape through the middle of the device to separate the individual twelve access channels. These channels were arranged in a grid array to enable drugs or signaling molecules flowing underneath to come into point contacts with the retina at specified locations. Since each access channel has its own inlet, outlet, and through-hole, different drugs or signaling molecules may be applied at the twelve different access points via through microscale apertures. The retina-on-a-chip scheme enabled study of spatially regulated, neuronal-glial interactions needed to examine tissue survival and response to pharmacology critical to both healthy and pathological retina.

## 5. Study of Developmental Processes

A fundamental goal of cell replacement therapy is to restore vision by using stem like cells to replace dysfunctional or damaged cells. The development of replacement cells has been transformed through the bioengineering manipulation of endogenous, somatic, and induced pluripotent stem cells [265,266]. However, successful stem cell transplantation is complex, as replacement cells must recapitulate key processes of retinogenesis to differentiate into retinal neurons, synapse with neighboring cells, and integrate within degenerated host retina to restore vision [105,113]. Microfluidic technologies have emerged as key systems to elucidate the guidance required for synapse formation, regulation of cell fate, and cellular alignment within complex structures.

### 5.1. Cone Growth and Guidance

Guidance of neuronal growth cones is an essential component of retinogenesis and regeneration, as these cellular extensions are the primary seekers of synaptic targets in neural tissues. Growth cone guidance of retinal neurons has been examined in microfluidic systems able to produce controllable gradients of extracellular molecules. One project produced a chip of microfluidic networks, called the microFN, to deliver different concentrations of the axonal guidance molecule, ephrinA5 [267]. The microdevice was comprised of an elastomeric stamp bonded onto a glass substrate whose transparency illustrated the growth cone response of developing retinal ganglion cells to ephrin gradients. A similar study used microfluidic channels to examine regeneration of outer cone segments of photoreceptor precursors in the presence of ciliary neurotrophic factor (CNTF) concentration fields [268]. Here, CNTF-secreting microdevices were implanted into one eye of mice at postnatal day 20, while control devices were implanted into the opposite eye. Sustained delivery of CNTF prevented cones from degeneration and helped maintain photoreceptor outer segments and light-sensing functions. A separate study applied microfluidic channels to investigate axon regeneration. Tests demonstrated that the mTOR pathway, a central regulator of mammalian metabolism [269], facilitated expression of axon pathfinding molecules to stimulate regeneration of human retinal ganglion cells after axotomy, i.e., axon severing [270]. These microfluidic projects have identified key extracellular factors able to stimulate and guide growth cones of retinal neurons to achieve the synaptic connectivity desired for regeneration and integration within damaged tissue.

### 5.2. Regulation of Cell Fate

Microfluidic perfusion has been applied to manipulate the diffusive signaling of cell-secreted factors that regulate the cell fate of mouse embryonic stem cells (mESCs). In one project [271], microfluidic chambers demonstrated differences in stem cell phenotype as a result of endogenous production of growth factors in the absence of perfusion (dilution), including Leukemia inhibitory factor (LIF), an interleukin cytokine that inhibits differentiation. At the protein level, mESCs produced over 140 times more LIF inside microchambers than in standard 2D culture, where changes in microchamber height (volume) were used to regulate pluripotent phenotype of stem cells [272]. A more recent study produced the first long-term, continuously perfused microfluidic system for retinal differentiation of human induced pluripotent stem cells (hiPSCs) [273]. The perfusion flow rate was established via measurement of consumption/degradation of key growth factors significant to neural differentiation and survival, including insulin-like growth factor (IGF-1), and FGF. Results of qRT-PCR analyses illustrated increased viability compared to cells cultured in multiwell plates, as well as increased expression of critical retinal differentiation markers, such as Rhodopsin. These data demonstrate that convective delivery of nutrients via perfusion has a significant impact on the expression of key retinal differentiation markers desired in replacement cells.

### 5.3. Cell Alignment and Positioning

Microtechnology has been similarly applied to study retinal differentiation using topographical cues. One study developed a biodegradable polycaprolactone (PCL) thin film scaffold with microfabricated topography to enhance the attachment and organization of retinal progenitor cells [274]. The project demonstrated that microscale topographical cues influenced cell attachment and differentiation of retinal progenitor cells independent of biochemical cues. Interestingly, while progenitor attachment to PCL topography resulted in the up-regulation of fate specific markers, no dramatic change in morphology was observed. These data were in contrast to a previous study where retinal progenitor cells grown on PCL thin films illustrated large changes in morphology [275,276]. An additional project [277] used the PCL scaffolds to demonstrate that retinal progenitor cells cultured on PCL exhibited enhanced potential to differentiate toward a photoreceptor fate in comparison to progenitors cultured on control substrates. Moreover, PCL constructs with embedded retinal progenitors demonstrated increased progenitor integration rates when co-cultured with retinas explanted from rhodopsin null mice. These promising contributions suggest that the scale of micropatterning on PCL scaffolds can guide differentiation of retinal progenitors towards a photoreceptor fate in vitro before transplantation.

Another project used soft lithography-patterned poly (lactic-co-glycolic acid) scaffolds (PLGA) to guide the morphogenesis of retinal cells derived from both human embryonic stem cells (hESCs) and dissociated newborn mouse retinas [199]. The project reconstructed retinal tissue by seeding dissociated cells into an array of aligned units that mimicked retinal anatomy. PLGA was solvent-processed into a microchannel scaffold format to achieve this geometric constraint. Results illustrated that rod photoreceptors and Müller glia aligned processes parallel to the microchannel walls, while sub-populations with retinal differentiation markers illustrated self-alignment between them. A separate project used PLGA to demonstrate that human iPSC-derived retinal ganglion cells exhibited an increased level of dendrite complexity within PLGA in vitro [278]. Retinal ganglion cells within the biomaterial were seen to extend dendrite-like and axon-like structures, whose morphology was more distinct that those observed using contemporary tests of ganglion cells on coverslips. Retinal ganglion cells were additionally observed to migrate into the scaffold and develop an axon hillock, i.e., part of the soma that connects to the axon, demonstrating abilities to exhibit distinct morphological character within PLGA scaffolds. These results are significant for biomaterial delivery of replacement cells, as cell morphology has been linked to endogenous cell repair and differentiation (as described in Section 4.2).

Micropatterned substrates were recently used to affect the differentiation and behavior of human neural progenitors in the developing retina [279]. Micropatterned polystyrene substrates were fabricated and coated with ECL (entactin, collagen, and laminin) to provide physical and chemical guidance during the differentiation of human neuronal progenitor cells (hNPCs). The hNPCs cultured on the micropatterned substrates were aligned and motile in the direction of the micropattern, compared with those cells growing on the non-patterned substrates. The hNPCs were then xeno-transplanted into the developing retina to study reciprocal interactions between the host tissue and the grafted cells. Cells displayed extensive survival, differentiation, and morphological integration following retinal xeno-transplant, even in the absence of immunosuppression. Moreover, morphological integration of grafted cells was most apparent in areas of retinal damage, indicating that multipotent hNPCs were capable of surviving and differentiating following culture upon micro-patterned substrates. These findings constitute an important advance in the development of tissue-level retinal models using microtechnology, which has enriched the study of developmental processes within adult retina. A different study used a motion-free bioreactor to culture retinal spheroids on a novel, micro-scaffold microfluidic device, called the cf-chip [280]. The device is constructed of PMMA (poly-methyl-methacrylate) bonded to a perforated PC (polycarbonate) membrane by a solvent welding-based process. The resulting cf-chip is comprised of 506 microfluidic compartments with characteristic dimensions on order of 100 microns, and houses millions of cells. The cf-chip is inserted into a custom-made bioreactor that is part of a closed circulation loop comprised of a roller pump and a medium reservoir connected to a gas mixing station. The bioreactor operates in so-called superfusion mode, where medium enters via the lower compartment and flows parallel to the membrane to the upper compartment to exit the bioreactor. Applications of this device produced individual retinal spheroids at high numbers from the postnatal retina, as observed for 10 days in vitro. The results illustrated spheroid size, internal structure, and cellular differentiation that were similar to retinal spheroids produced using conventional, rotation culture conditions. These contributions have enriched the testing of stem cell differentiation in a parallel platform, shown to promote integration in cell replacement therapies [103,281].

### 5.4. Stem Cell Delivery

The innovation of 3D microstructured scaffolds has dramatically altered delivery of replacement cells into adult retina [215,282]. Microfabrication techniques have been employed to create artificial tissue constructs comprising microfibers, microparticles, and hydrogel building blocks. Microfibers can be directly used as a scaffold, whereas hydrogel building blocks can be used to create tissue building blocks [283]. One study used blends of poly (L-lactic acid-co-glycolic acid)/poly (hydroxybutyrate-co-hydroxyvaleric acid) polymers (PLGA-PHBV8) as temporary scaffolds for transplantable photoreceptor precursors [284]. The results of this project have been the first to illustrate that micropatterned substrates can guide the laminar organization of transplanted cells. A complementing project then produced a 3D microstructure within elastomer films (biodegradable poly (glycerol-sebacate) and non-biodegradable poly (dimethyl) siloxane to serve as polarizable delivery scaffolds for photoreceptor precursors [285]. These microstructures consisted of an array of cup-shaped photoreceptor capture wells that funneled into a microchannel. This so-called wine glass scaffold design promoted efficient capture of hiPSC-derived photoreceptor cell bodies and guidance of basal axon extensions. The system provided significant contributions to cell replacement therapy because experiments ultimately achieved a uniform level of photoreceptor organization and polarization that has not been possible using conventional bolus injections or previously-described bio-scaffolds [215].

## 6. Future Directions

Microfluidic technologies have transformed contemporary understanding of retinal cell processes and helped apply this newfound knowledge to cultivate regenerative strategies for adult retinal disorders. Persistent challenges in cell replacement therapy, however, require new microfluidic models and bioassays.

### 6.1. Macular Models

One significant hindrance to retinal cell replacement is the lack of retinal models with similar critical characteristics of the human eye. While mouse models have produced outstanding contributions to bioengineering in the visual system, and beyond, the rodent retina is rich in rod photoreceptors used for night vision, but low in cone photoreceptors needed for visual acuity. Moreover, the rodent retina lacks a macula, where cone cells reside and most vision loss originates. Microfluidics, designed to create a cone-enriched environment, and/or macula-like structure, will greatly aid the study of cone pathology and degeneration to support integration during cell replacement therapy [113,211].

### 6.2. Retinal Blood Barriers

Significant microfluidic contributions to regenerative medicine can also be made via systems able to examine adult, collective cellular responses. Although, miniaturized bioassays have elucidated a wealth of behaviors from individual retinal cell groups (e.g., cone photoreceptors, amacrine cells), mechanisms of cooperative response between multiple neuronal groups, with and without glia, remain incompletely understood. Integrated cellular responses of mature retinal networks is especially significant to cell replacement therapy because endogenous neurons are likely to respond collectively to stem-like cells transplanted to replace dysfunctional components. Future directions for microfluidics also include the design of comprehensive cellular models for the retinal blood barrier, both the outer barrier formed at the RPE and the inner barrier at the retinal anterior. Although select microfluidic models of the retinal blood barrier exist [128,129,261], the projects focus on endothelial cells, and more recently pericytes, but fail to include Müller glia despite their critical roles in retinal metabolism and transport to/from the vasculature. Integration of Müller cell behaviors and neuroprotective responses elucidated using microfluidic bioassays will greatly improve microfluidic models used to validate and deliver pharmacology to treat retinopathies associated with chronic cardiovascular conditions or illness.

### 6.3. Hybrid Micro-Physiological Systems

Microfluidic technologies multiplexed into micro-physiological systems (MPS), or organoid models, have endowed transformative opportunities to examine and manipulate retinogenesis via genetic modifications, enriched extracellular matrix, and external cues and signaling fields, such as chemical gradients and electrical stimulus. However, these 3D models mimic developing retinal tissue produced using stem cells, which create substantially different cues than mature and/or degenerated retina in adults. Future MPS study is needed to incorporate adult retinal cells, such as Müller glia or ganglion cells, to develop hybrid, or blended, in vitro models able to guide emerging therapies for adult tissue more appropriately.

### 6.4. Microfluidic Testing Models

Lastly, the community is in need of novel microfluidic and macroscale systems to augment parallel testing models. While multi-well substrates have provided critical information about cell response to different external growth factors and pharma-compounds, microfluidic systems designed to manipulate and examine multiple explants or inoculated eyes remains underdeveloped. Such “assistive” microfluidic technologies will help empower translation of therapies from cellular samples to retinal explants, and organoid retinal models to adult retina. The examination of different responses from each biological component to the same stimulus will greatly elucidate the behaviors of adult tissues needed for regeneration.

In summary, while microfluidic contributions have bolstered a revolution in neuro-regenerative medicine, many opportunities remain to interrogate the specialized behaviors of mature cells and thecomplexity of adult cues from healthy and damaged tissue. Moreover, quantitative, microscale study of cellular interconnectivity between endogenous and de/differentiated replacement cells will significantly advance current understanding of thesequence and nature of synaptic integration processes needed for functional stem cell cell replacement.

## Abbreviations

AMD	Age-related Macular Degeneration (wet and dry)
CNTF	Ciliary Neurotrophic Factor
CTGF	Connective Tissue Growth Factor
Cx43	Gap junction protein Connexin 43
EGF	Epidermal Growth Factor
ESCs	Embryonic Stem Cells (mouse)
FGF	Fibroblast Growth Factor
GCL	Ganglion Cell Layer of Retina
GDNF	Glia-Derived Neurotrophic Factor
IGF	Insulin-like Growth Factor
INL	Inner Nuclear Layer of Retina
iPSCs	Induced Pluripotent Stem Cells (human)
I/R	Ischemia-Reperfusion Injury Model
MPS	Micro-Physiological Systems (or organoids)
MSCs	Mesenchymal Stem Cells
NrF2	Nuclear Factor Erythroid 2-related factor 2
NS	Nervous System (Central and Peripheral)
ONL	Outer Nuclear Layer of Retina
PDMS	Poly (dimethyl) siloxane
PCL	Polycaprolactone
PDGF	Platelet-Derived Growth Factor
PEDF	Pigment Epithelium-Derived Factor
PEM	Polyelectrolyte Multilayer
PLGA	Poly (lactic-co-glycolic acid)
PMMA	Poly (methyl-methacrylate)
SDF-1	Stromal-Derived Growth Factor
TGF-b	Transforming Growth Factor Beta
VEGF	Vascular Endothelial Growth Factor
WHO	World Health Organization
